# Supplementary material for: Proteomic Insights into the Interaction of Chitosan Nanoparticles with Canine MDCK Epithelial Cells
Source: Molecules. 2025 Sep 19;30(18):3815. doi: 10.3390/molecules30183815 (PMC12473073; doi:10.3390/molecules30183815)
Supplement: Supplementary file 1 [file molecules-30-03815-s001.zip › molecules-3755328-supplementary.pdf]

Supplementary Material

Section S1. In our work, three average nanoparticle sizes are reported. Nanoparticles were spontaneously formed by incorporating a TPP solution into a CS solution. Micrographs (Figure S1) obtained by atomic force microscopy of nanoparticle sizes of 15 and 30 nm.

The grain analysis was developed by employing the software from the AFM brand (NTMDT) of the chitosan nanoparticles (Figure S2), which determines the particle size is also integrated, establishing an average size of 16, 34, and 127 nm, respectively. The results were captured in other software and are shown in Figure S1.

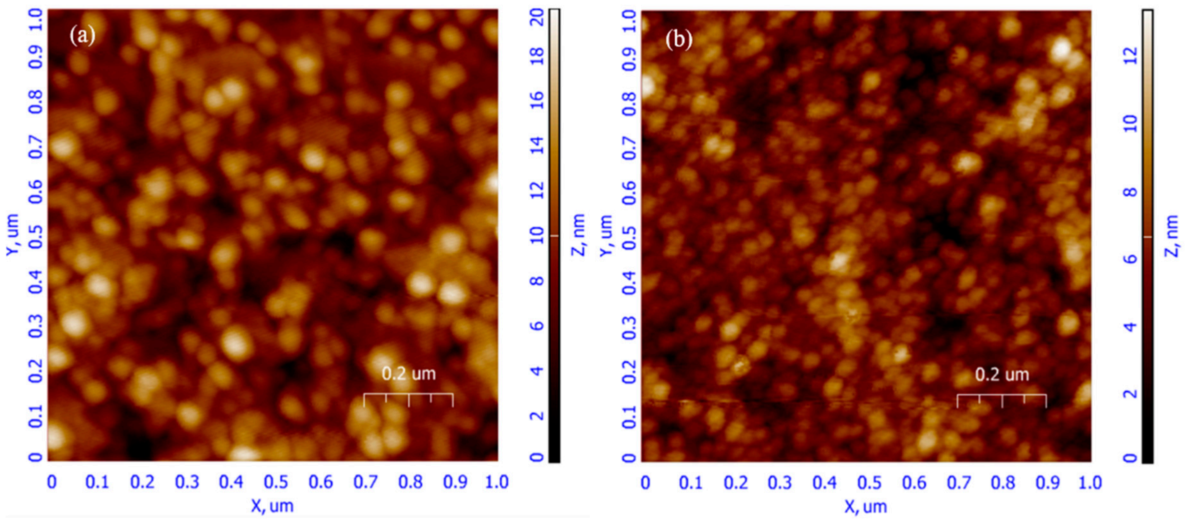

Figure S1. Micrographs correspond to the <30 nm> (a) and <15 nm> (b) protocols of the chitosan nanoparticles.

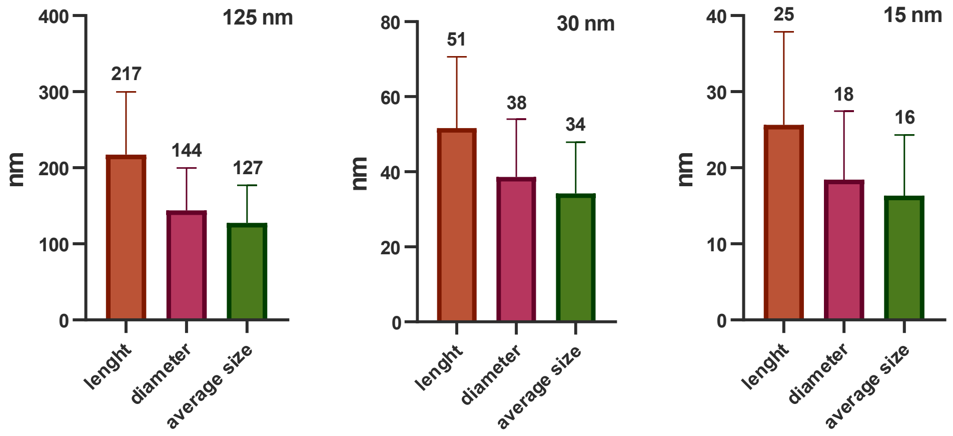

Figure S2. Quantitative graphs of the grain analysis of chitosan nanoparticles.

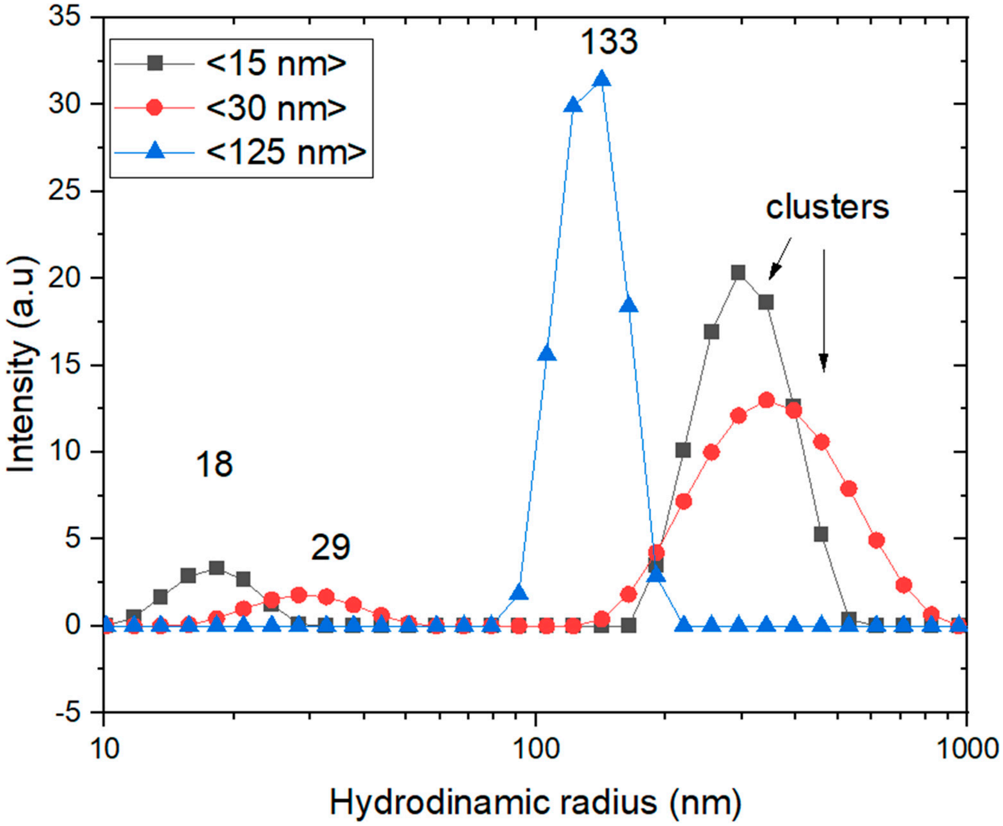

Figure S3. Dynamic Light Scattering analysis of chitosan nanoparticles for the three different protocols.

Section S2. Figure S4 shows the areas where the wound was made to the monolayer, establishing with black dotted lines the edge of the initial width of the wound. The result demonstrated that chitosan nanoparticles (125 nm) with a concentration of 400 µg/ml promote cell motility, where complete closure of the wound was observed after 8 h compared to the control condition. To make the healing effect of chitosan more evident, immunofluorescence assays of wound closure were performed with the two highest concentrations of chitosan nanoparticles (300 and 400 µg/ml), showing a tracking of the wound length and width by confocal microscopy. Figure S5 shows a reconstruction of five fields at 10x of each treatment plus the control.

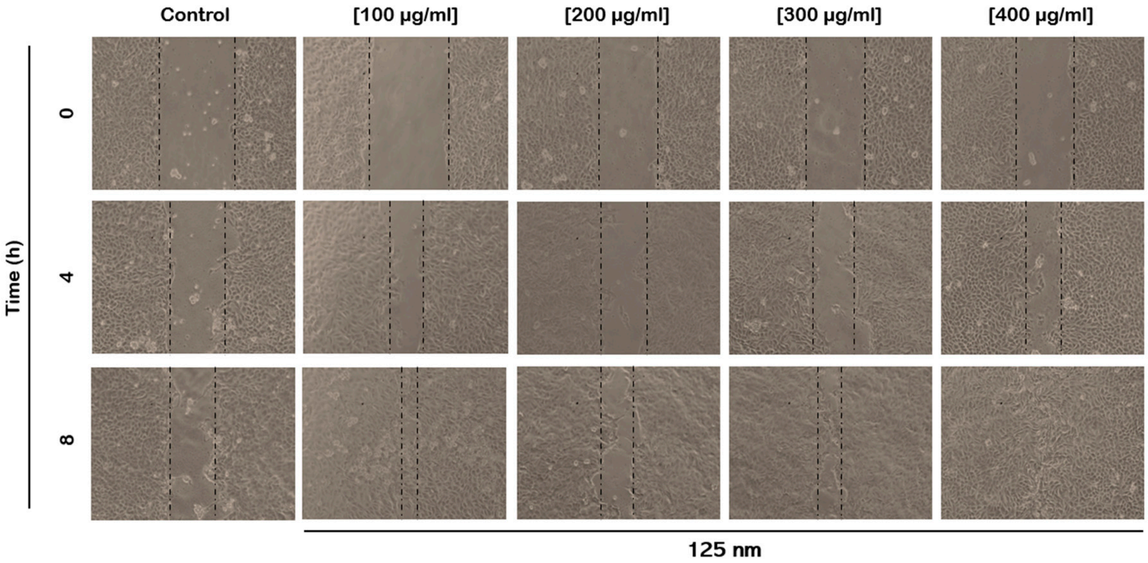

Figure S4. Representative bright field images of the wound-closure assay. The photographs were taken with a NIKON camera at 5 s shutter opening. This image is a reconstruction of different biological replicates of the assay (n=3).

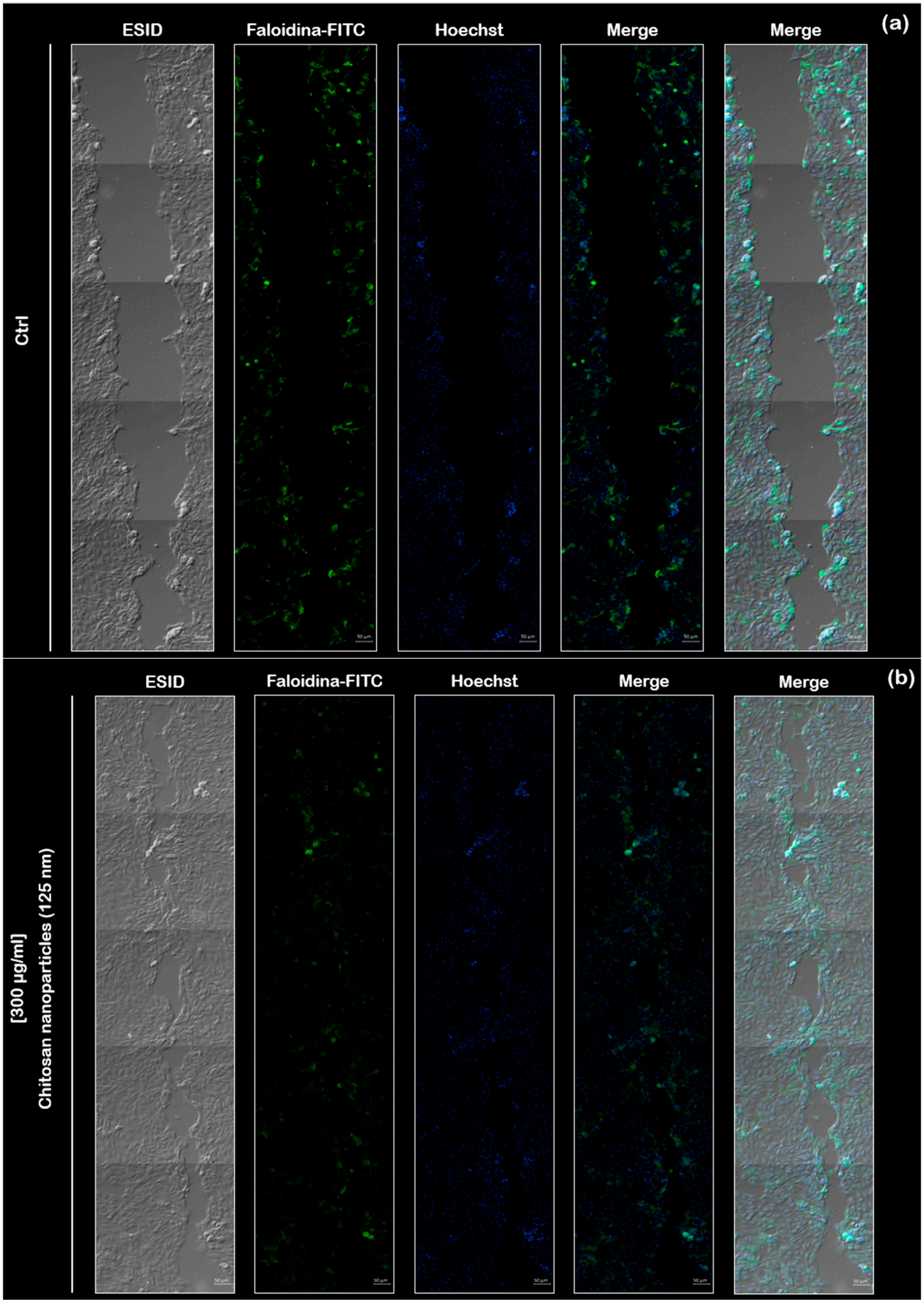

(a)
Ctrl
ESID
Faloidina-FITC
Hoechst
Merge
Merge
(b)
[300 µg/ml]
Chitosan nanoparticles (125 nm)
ESID
Faloidina-FITC
Hoechst
Merge
Merge

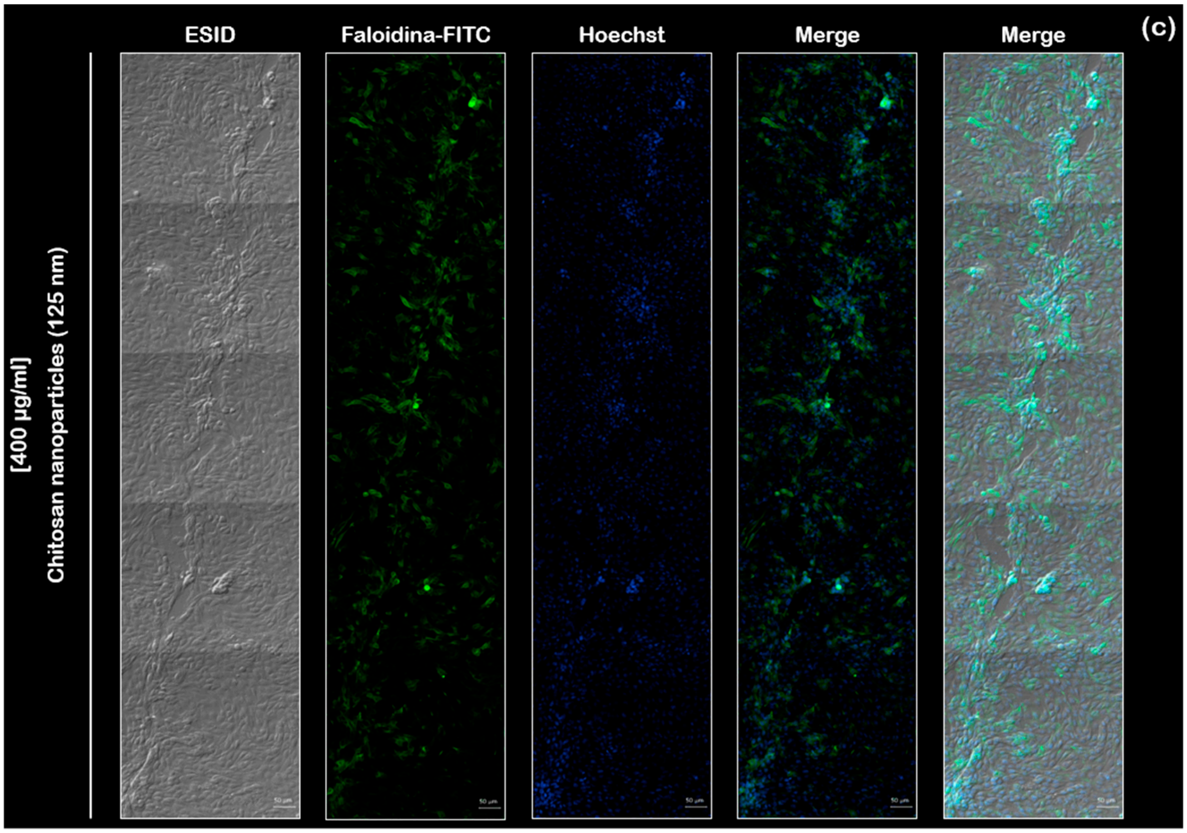

Figure S5. Images obtained by confocal microscopy (50 µm) of the wound closure assay: (a) control, (b) chitosan nanoparticles at 300 µg/ml, and (c) chitosan nanoparticles at 400 µg/ml. Staining of the cytoskeleton by FITC-coupled Phalloidin and nuclei stained in blue by Hoechst, with their respective merge; also, the bright field images (ESID) and the merge of the coupling between ESID, cytoskeleton, and nucleus.

Section S3. This section presents the wound closure assay using 15 nm CS NPs. Figure S6 illustrates the dynamics of wound closure as a function of cell migration. The results indicate that the 15 nm nanoparticles did not promote cell migration after 12 hours of treatment, compared to the control group.

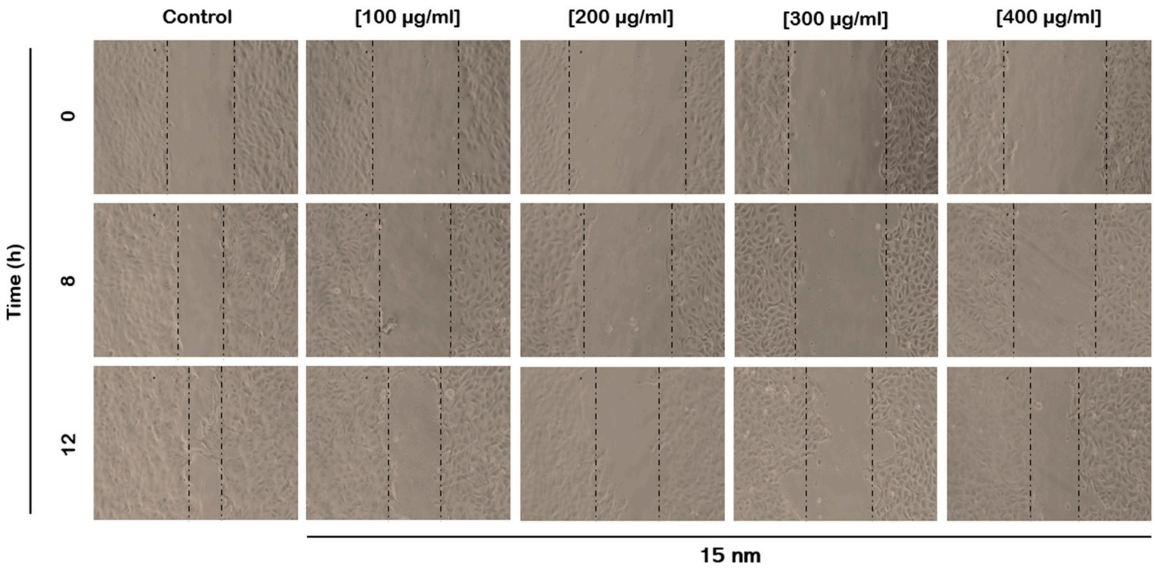

Figure S6. Representative bright field images of the wound-closure assay of CS NPs (15 nm). The photographs were taken with a NIKON camera at 5 s shutter opening. This image is a reconstruction of different biological replicates of the assay (n=3).
